# Contribution to More Patient-Friendly ART Treatment: Efficacy of Continuous Low-Dose GnRH Agonist as the Only Luteal Support—Results of a Prospective, Randomized, Comparative Study

**DOI:** 10.1155/2015/727569

**Published:** 2015-04-05

**Authors:** Céline Pirard, Ernest Loumaye, Pascale Laurent, Christine Wyns

**Affiliations:** ^1^Department of Gynecology-Andrology, Cliniques Universitaires Saint-Luc, Institut de Recherche Expérimentale et Clinique (IREC), Université Catholique de Louvain, 1200 Brussels, Belgium; ^2^ObsEva SA, 1228 Geneva, Switzerland

## Abstract

*Background*. The aim of this pilot study was to evaluate intranasal buserelin for luteal phase support and compare its efficacy with standard vaginal progesterone in IVF/ICSI antagonist cycles. *Methods*. This is a prospective, randomized, open, parallel group study. Forty patients underwent ovarian hyperstimulation with human menopausal gonadotropin under pituitary inhibition with gonadotropin-releasing hormone antagonist, while ovulation trigger and luteal support were achieved using intranasal GnRH agonist (group A). Twenty patients had their cycle downregulated with buserelin and stimulated with hMG, while ovulation trigger was achieved using 10,000 IU human chorionic gonadotropin with luteal support by intravaginal progesterone (group B). *Results*. No difference was observed in estradiol levels. Progesterone levels on day 5 were significantly lower in group A. However, significantly higher levels of luteinizing hormone were observed in group A during the entire luteal phase. Pregnancy rates (31.4% versus 22.2%), implantation rates (22% versus 15.4%), and clinical pregnancy rates (25.7% versus 16.7%) were not statistically different between groups, although a trend towards higher rates was observed in group A. No luteal phase lasting less than 10 days was recorded in either group. *Conclusion*. Intranasal administration of buserelin is effective for providing luteal phase support in IVF/ICSI antagonist protocols.

## 1. Introduction

Cycles resulting from controlled ovarian hyperstimulation (COH), when downregulated with gonadotropin-releasing hormone (GnRH) agonist or GnRH antagonist, commonly result in luteal phase deficiency [1, 2]. This phenomenon is characterized by low progesterone levels, delayed endometrial secretory transformation, and a shortened luteal phase of less than ten days [3], resulting in reduced embryo implantation, lower pregnancy rates, and increased miscarriage rates. For this reason, luteal phase support is a common practice in IVF treatments, as it significantly improves embryo implantation, pregnancy, and delivery rates [2].

Vaginal progesterone with or without estradiol and intramuscular human chorionic gonadotropin (hCG) are the current regimens used for luteal phase support. Since hCG administration is associated with the risk of ovarian hyperstimulation syndrome (OHSS), progesterone is the preferred choice [4].

It was reported that GnRH agonists administered during the luteal phase in addition to standard luteal phase support improved pregnancy rates in IVF-stimulated cycles [5–14], but the mechanism of this apparently beneficial effect is poorly understood. GnRH agonists may support the corpus luteum by stimulating the secretion of luteinizing hormone (LH) by pituitary gonadotroph cells, by acting directly on the endometrium through locally expressed receptors, or by their direct effect on the embryo [6, 7].

The question as to whether GnRH agonist alone is able to efficiently support the luteal phase nevertheless remains.

We previously reported that, in patients undergoing COH, intranasal (IN) administration of buserelin for 15 days is able to trigger final follicular maturation and has a marked, dose-related effect on the luteal phase [15]. This observation led to the conclusion that the optimal dose of buserelin for luteal support in an antagonist protocol is 100 *μ*g 3 times per day.

To further analyze the potential benefits of this new protocol, our study objective was to assess the efficacy of luteal phase GnRH agonist administration in cycles where ovulation was triggered by GnRH agonist and compare it with a standard protocol using hCG to trigger ovulation, followed by intravaginal progesterone as luteal support. To the best of our knowledge, this is the first randomized controlled trial using GnRH agonist as the only luteal phase support.

## 2. Materials and Methods 

### 2.1. Study Design and Patient Randomization

In order to study the effect of IN administration of GnRH agonist to trigger and support the luteal phase, we initiated a single-center, prospective, randomized, open, parallel group study. We were looking to compare this method in patients undergoing IVF/ICSI after stimulation of multiple follicular development with human menopausal gonadotropin (hMG). Inclusion criteria were the age between 18 and 39 and BMI ≥ 18 but ≤35, while exclusion criteria were a history of poor response, systemic disease (diabetes, severe migraine, hepatic, renal, or cardiovascular disease, and corticodependent asthma), and ovarian cysts ≥11 mm.

Computer-generated randomization was applied (2/1; group A/B). Treatment allocation instructions were placed in individually sealed envelopes to be opened at the center in chronological order on the day of signing the informed consent form.

In study group A, GnRH agonist (buserelin) was administered IN to trigger final follicular maturation and support the luteal phase. In control group B, hCG was administered to trigger final follicular maturation and vaginal progesterone to support the luteal phase.

The primary end-point was the comparison of pregnancy rates between the two groups.

The study protocol and informed consent form were approved by the institution's ethics committee.

### 2.2. Ovarian Stimulation Protocol and Treatment Groups

**Figure 1:**
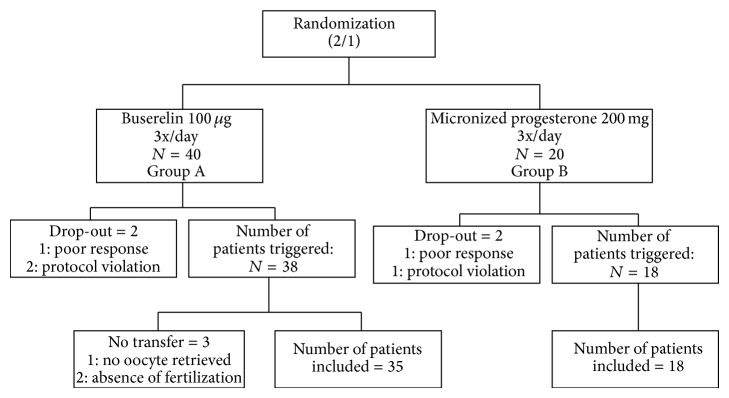
Randomization and allocation of patients to the two groups. In group A, ovulation was triggered with buserelin and the luteal phase was also supported by buserelin. In group B, ovulation was triggered with hCG and the luteal phase was supported with vaginal micronized progesterone.

Sixty patients with infertility factors indicating IVF or ICSI were enrolled in this study, as shown in Figure 1.

In study group A, 40 patients underwent COH using hMG (Menopur, Ferring, Brussels, Belgium) at a dose ranging from 150 to 450 IU daily. When the leading follicle reached a mean diameter of 14 mm, daily administration of 0.25 mg GnRH antagonist (Orgalutran; MSD, Brussels, Belgium) was initiated every evening up to the day before ovulation trigger.

When patients met the criteria for ovulation trigger (at least 3 follicles >17 mm), they received 200 *μ*g of IN buserelin (Suprefact; Aventis, Brussels, Belgium), followed by 100 *μ*g IN buserelin three times a day for luteal support starting the next day, as previously published by our team [15]. This was continued for a maximum of 16 days until the day of the pregnancy test, whether or not it proved positive.

In control group B, 20 patients had a cycle downregulated with GnRH agonist and stimulated with hMG at a dose ranging from 150 to 450 IU daily. When they met the criteria for ovulation trigger (at least 3 follicles >17 mm), they were given 10,000 IU subcutaneous hCG (Pregnyl; MSD, Brussels, Belgium), followed by 200 mg vaginal progesterone three times a day (Utrogestan; Goodlife pharma, Lelystad, Netherlands), starting on the day of oocyte pick-up for luteal support. Progesterone was administered up to day 16 (day of the pregnancy test). If the pregnancy test proved positive, progesterone administration was continued up to week 12 of pregnancy, which is a common practice in our center.

Pregnancy was diagnosed by measuring serum hCG levels on day 14 of the luteal phase (day of first hCG/buserelin administration = D0). A pregnancy test was considered positive if an increase in serum hCG was observed after a first test showing at least >10 mIU/mL. The implantation rate was calculated as the number of gestational sacs divided by the number of transferred embryos.

Clinical pregnancy was defined as the presence of an intrauterine gestational sac with a positive heartbeat visualized by vaginal ultrasound.

The duration of the luteal phase was calculated from day 1 (the first day after ovulation trigger) up to the day before menstruation commenced.

IVF laboratory culture conditions were the same as those previously described [15].

All embryos were transferred on day 3.

### 2.3. Hormone Assays

Serum estradiol, serum progesterone, and serum LH concentrations were monitored on D0 (day of ovulation trigger), D2, D5, D9, and D14. Hormone levels were determined with commercially available kits routinely used in our accredited clinical center's central laboratory. Estradiol, progesterone, and LH were assayed using the Elecsys 2010 system (Roche Diagnostics GmbH, 68298 Mannheim, Germany). The E2 intra-assay coefficient of variation (CV) was <6% and the interassay CV was <6%; the P4 intra-assay CV was <3% and the interassay CV was <6%; the LH intra-assay CV was <2% and the interassay CV was <5%. Serum hCG was assayed using the Beckman-Coulter system (Anablis, Namur, Belgium), yielding an intra-assay CV of <2% for a value <5 mIU/mL and an interassay CV of <12%.

### 2.4. Statistical Analysis

Descriptive quantitative statistics (mean and SD) were calculated for demographic and ovarian stimulation status parameters, IVF/ICSI and embryo transfer parameters, and luteal phase duration. Comparison between groups was done using the student *t*-test. *P* < 0.05 was considered statistically significant.

## 3. Results 

Sixty patients were randomized at a ratio of 2/1 (group A/group B). Five patients dropped out of group A and two from group B, leaving 53 patients eligible for analysis. Patient groups did not differ in their basic demographic characteristics, and COH parameters were also comparable (Table 1).

The mean number of retrieved oocytes, cleaved embryos, and transferred embryos did not show any statistical difference. Embryo transfer was performed in both groups, with a mean number of 1.4 embryos per transfer (Table 2). Furthermore, the mean number of good quality embryos was similar in both groups.

In group A, 11 positive pregnancy tests were recorded during the study. Nine pregnancies were confirmed as clinical pregnancies, 7 of which were singleton and 2 were twin pregnancies. One pregnancy ended in miscarriage at 9 weeks and one was terminated because of spina bifida and another for severe preeclampsia. Eight healthy babies were born after 37 weeks of pregnancy.

In group B, 4 positive pregnancy tests were recorded during the study. Three pregnancies were confirmed as clinical pregnancies, 2 of which were singleton and one was a twin pregnancy. Four healthy babies were born after 37 weeks of pregnancy.

While similar numbers of retrieved oocytes and transferred embryos were found between groups, patients in group A showed a tendency towards a higher pregnancy rate (31.4% versus 22.2%), implantation rate (22% versus 15.4%), and clinical pregnancy rate (25.7% versus 16.7%), despite the fact there is no statistically significant difference (Table 2).

Endometrial thickness on D9 of the luteal phase was also similar in both groups (10.7 ± 3 mm versus 10.5 ± 4.4 mm).

**Figure 2:**
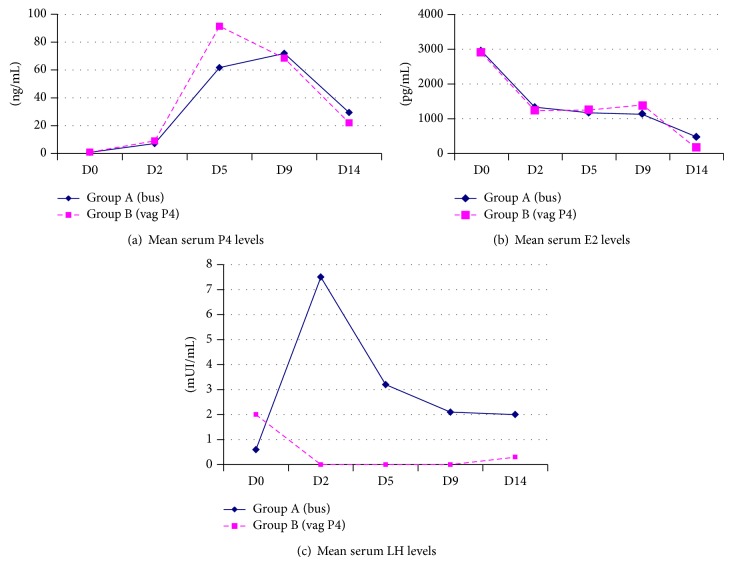
Hormone levels during the luteal phase on D0 (day of ovulation trigger), D2, D5, D9, and D14. (a) Mean progesterone levels. (b) Mean estradiol levels. (c) Mean LH levels.

Hormone levels during the luteal phase are shown in Figure 2. The progesterone profile during the luteal phase was similar in both groups, except on D5, where it was significantly higher (*P* < 0.05) in group B (91.4 ± 38.8 ng/mL) than in group A (61.7 ± 36.9 ng/mL) (Figure 2(a)). Estradiol levels were comparable during the entire luteal phase (Figure 2(b)). By contrast, mean serum LH levels were significantly higher throughout the luteal phase in group A than in group B (where serum LH levels were below the limit of detection (<0.1 IU/L) in most samples taken between D2 and D9) (Figure 2(c)).

The duration of the luteal phase assessed in patients who did not become pregnant ranged between 11 and 21 days in group A (mean 13.8 ± 2.3) and between 11 and 19 days in group B (mean 14.7 ± 2.7). No luteal phase lasting less than 10 days was recorded in either group.

Buserelin treatment was well tolerated by all patients. There was no further drop-out, and no significant adverse events were reported in terms of local or systemic tolerance. No OHSS requiring hospitalization was recorded.

## 4. Discussion 

During the menstrual cycle, a normal luteal phase is required for embryo implantation and evolution of pregnancy. The luteal phase is the result of intermittent stimulation of the corpus luteum by pituitary LH. During the luteal phase, pituitary LH pulses are of low frequency, leading to extended episodes of progesterone secretion at a rate of 3–5 per 24 hours [20]. Luteal phase deficiency is a common feature of cycles resulting from COH [21] including GnRH antagonist-treated cycles [22–24]. It is characterized by premature regression of the corpus luteum, leading to a shortened luteal phase (<10 days), low serum progesterone levels, and delayed secretory transformation of the endometrium [25]. Consequences of luteal phase deficiency are reduced embryo implantation rates, lower pregnancy rates, and increased miscarriage rates when pregnancy is established [2]. Besides standard luteal phase support with progesterone, various other methods have also been studied, including use of low-dose hCG [26–30], hCG according to ovarian response to stimulation [31], recombinant LH [32], and intensive progesterone and estradiol administration [33–35]. Because inadvertent administration of GnRH agonist does not appear to compromise pregnancy outcome [36], a number of studies have also investigated the potential benefits of using GnRH agonist for luteal phase support in IVF/ICSI cycles (Tables 3 and 4). The findings are somewhat contradictory, however, with studies identifying a positive [5, 7, 9, 10, 13], absent [12, 14, 16], or negative [17, 19] impact on outcome in long agonist stimulation protocols. Although the number of studies investigating antagonist protocols is smaller, a similar positive influence on implantation and pregnancy rates has been reported in most of these [7, 11, 13, 15]. Protocols of GnRH agonist administration vary, with intermittent single, double, or multiple doses or continuous administration during the luteal phase. It is noteworthy that all studies use GnRH agonist in combination with other luteal phase support methods previously mentioned. No comparison can therefore be made between our results and those in the reported literature. In our previous dose-finding study on the use of GnRH agonist for luteal support, we demonstrated that buserelin is able to induce final follicular maturation, trigger ovulation, and support alone the luteal phase when administered at the appropriate dose [15].

Luteal phase support after COH is even more important when triggering with GnRH agonists. Previous randomized controlled trials [37, 38] found that the use of GnRH agonist to trigger ovulation was associated with negative clinical results, namely, low implantation and clinical pregnancy rates and high rates of early pregnancy loss, presumably related to luteal phase insufficiency despite standard supplementation with progesterone and estradiol [27]. Only intensive progesterone and estradiol support [33–35] and hCG [26–31] or recombinant LH [32] were able to normalize the luteal phase after GnRH agonist administration for ovulation trigger. Finally, in a very recent Cochrane review, Youssef et al. [39] reported that, in women undergoing fresh autologous IVF/ICSI cycles, GnRH agonists were associated with a lower ongoing pregnancy rate than that obtained with hCG (OR = 0.70; 95% CI: 0.54 to 0.91). However, the effect was dependent on the type of luteal support provided. The higher pregnancy rate in the hCG trigger group applied only to the GnRH agonist trigger group that received luteal support without LH activity (OR = 0.36, 95% CI: 0.21 to 0.62) [39].

Studies showing that implantation rates remain normal in oocyte recipients and frozen-thawed cycles with embryos issuing from protocols with GnRH agonist trigger [40, 41] confirm that a deficient luteal phase is the main problem leading to reduced outcomes.

In our study, similar outcome parameters were obtained when GnRH agonist was used to trigger ovulation, demonstrating that GnRH agonist (IN buserelin) is able to adequately support the luteal phase. Indeed, the implantation potential of our embryos did not appear to be hampered compared to the standard protocol using hCG for ovulation trigger and vaginal progesterone as luteal support. Furthermore, an additional study conducted in our department, in which hCG was used to trigger ovulation and GnRH agonist (without any other supplementation) to support the luteal phase, found similar pregnancy rates (data not shown) and hence corroborates the current study.

The beneficial effect of GnRH agonist during the luteal phase may be linked to its impact on the embryo or a direct or indirect effect on the endometrium. Indeed, GnRH receptors have been shown to be present in preimplantation human embryos in the luteal phase at both the mRNA and protein levels [42, 43], and different observations support the hypothesis that GnRH agonist exerts a direct beneficial effect on embryos [6, 7]. Animal experiments suggest that GnRH agonists can enhance the in vitro development of embryos [42, 44–46]. In addition, GnRH agonists appear to have a regulatory impact on the synthesis and secretion of hCG by preimplanted embryos and the placenta [7, 47, 48].

Direct action on uterine tissue may also be responsible for the effects of GnRH agonists in the luteal phase. The presence of a GnRH receptor showing a dynamic pattern (more intense in the luteal phase) was demonstrated in human endometrium, both in the epithelium and stroma, providing evidence that GnRH may play a key role as a molecular autocrine-paracrine regulator in embryonic-endometrial interactions during early implantation [49–51].

The corpus luteum is another possible GnRH agonist target, though it is questionable whether such action would occur through the secretion of pituitary hormones or by direct action in the ovary [52]. In cycles with GnRH antagonists, it is speculated that the stimulation of corpus luteum activity by GnRH agonist may result from the stimulation of LH secretion, given that, despite the blockade, the pituitary gland remains responsive to GnRH or GnRH agonist [11].

In our study, we found completely different LH profiles during the luteal phase between the two groups, with significantly higher LH levels when GnRH agonist was administered for luteal support. Although preliminary, our data suggest that maintaining LH secretion throughout the luteal phase by repeated administration of GnRH agonist could overcome the drawbacks of GnRH agonist-induced final follicular maturation followed by standard luteal support.

Besides LH-mediated stimulation of steroid production by the corpus luteum, LH activity could also have an impact on the endometrium through LH receptors expressed in the endometrium [53–55] or by promoting expression and secretion of relaxin [56], angiogenic and growth factors, and cytokines involved in implantation [57].

*In conclusion*, our study confirms that GnRH agonist is able to trigger ovulation and support the luteal phase in antagonist IVF cycles, showing comparable efficacy to the standard protocol. Furthermore, this new approach has several advantages over other forms of luteal support in terms of convenience for the patient, because nasal administration is easily done anywhere, is not painful, and does not require the help of a nurse. Since GnRH agonist was the only drug administered for luteal support, compared to other protocols that add GnRH agonist to their classic luteal support, this new protocol can contribute to more patient-friendly ART.

However, although all babies born in this study were in good health, our data need to be corroborated by larger series, and caution should still be exercised concerning the condition and state of health of children issuing from this protocol.

## Figures and Tables

**Table 1:** Patient demographics and stimulation parameters (mean ± SD).

	Group A (buserelin 3x/day) *n* = 35	Group B (micronized progesterone 3x/day) *n* = 18	*P*
Age (years)	32 ± 4.4	33 ± 4.5	NS
Range	2 ± 1.1	2 ± 1.3	NS
HMG (IU)	2526 ± 988	2614 ± 1077	NS
Day of trigger (=D0)	12.7 ± 2.7	12.9 ± 2.6	NS
Estradiol D0 (pg/mL)	2960 ± 1068	2929 ± 1439	NS
Progesterone D0 (ng/mL)	0.9 ± 0.4	1 ± 0.4	NS
Endometrial thickness D0 (mm)	9.8 ± 2.1	9.0 ± 3.0	NS

**Table 2:** IVF/ICSI cycle outcomes (mean ± SD).

	Group A (buserelin 3x/day) *n* = 35	Group B (micronized progesterone 3x/day) *n* = 18	*P*
Retrieved oocytes (*n*)	10.1 ± 4.1	10.7 ± 5.9	NS
Cleaved embryos (*n*)	5.8 ± 2.7	6.0 ± 3.2	NS
Transferred embryos (*n*)	1.4 ± 0.5	1.4 ± 0.5	NS
Pregnancy rate/transfer	11/35 31.4%	4/18 22.2%	NS
Clinical pregnancy rate	9/35 25.7%	3/18 16.7%	NS
Implantation rate	11/50 22%	4/26 15.4%	NS

**Table 3:** Main characteristics of trials on the use of a single injection of GnRH agonist for luteal phase support.

Trial	Study design	Stimulation protocol	Ovulation trigger	Luteal phase support			Day of ET	Implantation rate (study group/control group)	(Ong/Clin) Pregnancy rate (study group/control group)
				Study group GnRH-a	Control group	Other concomitant medications (all patients)			
Tesarik et al. [7]	RCT	GnRH-a long GnRH-antag	250 *µ*g r-hCG	*n*: 150 GnRH-a long *n*: 150 GnRH-antag 0.1 mg/triptorelin Single injection D6 after OPU	*n*: 150 GnRH-a long *n*: 150 GnRH-antag Placebo	Vaginal micronized progesterone (400 mg/d) + estradiol valerate (4 mg/d) + r-hCG (250 *µ*g; single dose on d of ET)	Day 3	GnRH-a long: 29.8% 18.2% (*P* < 0.05) GnRH-antag: 27.1% 17.4% (*P* < 0.05)	GnRH-a long: Ong PR/Tf: 46.8% 38.0% (NS) GnRH-antag: Ong PR/Tf: 44.8% 31.9% (*P* < 0.05)

Ata et al. [16]	RCT	GnRH-a long	10.000 IU u-hCG	*n*: 285 0.1 mg/triptorelin Single injection D6 after OPU	*n*: 285 Placebo	Vaginal micronized progesterone gel (90 mg/d)	Day 3	21.1% 20.1% (NS)	Ong PR: 31.2% 29.5% (NS)

Isik et al. [11]	RCT	GnRH-antag	10.000 IU u-hCG or 250 *µ*g r-hCG	*n*: 74 0.5 mg/leuprolide Single injection D6 after OPU	*n*: 80 No placebo	Vaginal micronized progesterone (600 mg/d) + hCG (single dose)	Day 3	26.5% 9.3% (*P* < 0.0001)	Clin PR: 40.5% 20.0% (*P* < 0.01)

Razieh et al. [10]	RCT	GnRH-a long	10.000 IU u-hCG	*n*: 90 0.1 mg/triptorelin Single injection D5 or D6 after OPU	*n*: 90 Placebo	Vaginal micronized progesterone (800 mg/d)	Days 2-3	12.3% 7.3% (*P* < 0.05)	Clin PR: 25.5% 10.0% (*P* < 0.05)

Ata and Urman [17]	RCT	GnRH-antag	10.000 IU u-hCG	*n*: 38 0.1 mg/triptorelin Single injection D6 after OPU	*n*: 52 Placebo	Vaginal micronized progesterone gel (90 mg/d)	Day 3	14.12% 27.27% (*P* < 0.05)	Ong PR: 18.4% 42.31% (*P* < 0.05)

Kung et al. [13]	Retrospective study	GnRH-a long GnRH-antag	500 *µ*g r-hCG	*n*: 147 0.1 mg/triptorelin Single injection D6 after OPU	*n*: 93 No GnRH-a	Vaginal micronized progesterone gel (90 mg/d) or i.m. progesterone (25–50 mg/d)	Days 3–5	24.5% 17.0% (*P* < 0.05)	Clin PR: 49.0% 33.3% (*P* < 0.05)

Yıldız et al. [14]	RCT	GnRH-a long	10.000 IU u-hCG	*n*: 100 1 mg/leuprolide acetate Single injection D6 after OPU	*n*: 95 No placebo	Vaginal micronized progesterone (600 mg/d) + 17*β* estradiol (4 mg/d)	Day 3	20.7% 13.3% (NS)	Ong PR: 36.0% 27.4% (NS)

**Table 4:** Main characteristics of trials on the use of multiple doses of GnRH agonist for luteal phase support.

Trial	Study design	Stimulation protocol	Ovulation trigger	Luteal phase support			Day of ET	Implantation rate (study group/control group)	(Ong/Clin) Pregnancy rate (study group/control group)
				Study group GnRH-a	Control group	Other concomitant medications (all patients)			
Fujii et al. [5]	RCT	GnRH-a long	5.000 IU u-hCG	*n*: 161 Continuous 600 *µ*g/d IN buserelin twice daily during the luteal phase until D14 after OPU	*n*: 158 No placebo	Dydrogesterone for 14 days (10 mg/d) + i.m. hCG on the day of ET (2.500 IU)	Days 2-3	28.5% 19.6% (*P* < 0.05)	Clin PR: 44.5% 34.3% (NS)

Pirard et al. [15]	RCT (dose finding study)	GnRH-antag	IN buserelin (study groups) 10.000 u-hCG (control group)	IN buserelin during the luteal phase until D14 after OPU 1 every 2 d (*n*: 2) 1/d (*n*: 3) 2/d (*n*: 6) 3/d (*n*: 6)	*n*: 6 Vaginal micronized progesterone (600 mg/d)	No other drug (for study groups)	Day 3	NA	NA

Hugues et al. [18]	ESHRE abstract	GnRH-antag	250 *µ*g r-hCG	*n*: 25 0.1 mg/triptorelin Two injections (D3 and D6 after OPU)	*n*: 22 No placebo	Vaginal micronized progesterone (400 mg/day)	Day 3	NA	NA

Isikoglu et al. [19]	RCT	GnRH-a long	10.000 IU u-hCG	*n*: 90 Continuous 0.25 mg/d leuprolide acetate during the luteal phase until 14 days after OPU	*n*: 91 No placebo	i.m. progesterone (50 g/day)	NA	35.6% 35.3% (NS)	Clin PR: 50.0% 52.32% (NS)

Qublah et al. [9]	RCT	GnRH-a long	10.000 IU u-hCG	*n*: 60 0.1 mg/triptorelin Three injections (D of OPU, D3, and D6 after OPU)	*n*: 60 Placebo	Vaginal progesterone (pessaries: cyclogest)	Day 3	21.4% 7.3% (*P* < 0.01)	PR: 36.6% 13.3% (*P* < 0.01)

Inamdar and Majumdar [12]	RCT	GnRH-a long	250 *µ*g r-hCG	*n*: 213 1 mg/leuprolide acetate Three injections (D6, D7, and D8 after OPU)	*n*: 213 No placebo	Vaginal progesterone (400 mg twice daily) alternating with i.m. natural micronized progesterone (100 mg) starting from the day of OPU	Day 2	17.57% 17.07% (NS)	Ong PR: 27.69% 26.29% (NS)

Yıldız et al. [14]	RCT	GnRH-a long	10.000 IU u-hCG	*n*: 84 1 mg/leuprolide acetate Two injections D6 and D9 after OPU	*n*: 95 No placebo	Vaginal micronized progesterone (600 mg/d) + 17*β* estradiol (4 mg/d)	Day 3	25.8% 13.3% (NS)	Ong PR: 42.9% 27.4% (NS)

d: day; ET: embryo transfer; GnRH-a: GnRH agonist; GnRH-antag: GnRH antagonist; IU: international unit; *n*: number of cycles; OPU: oocyte pick-up; r: recombinant; u: urinary; PR: pregnancy rate; Clin PR: clinical pregnancy rate; Ong PR: ongoing pregnancy rate; NA: not available; NS: not significant.
